# Multidetector-row computed tomography for evaluating the branching angle of the celiac artery: a descriptive study

**DOI:** 10.1186/1471-2342-12-36

**Published:** 2012-12-21

**Authors:** Hiroyuki Tokue, Azusa Tokue, Yoshito Tsushima

**Affiliations:** 1 Department of Diagnostic and Interventional Radiology, Gunma University Hospital, 3-39-22 Showa-machi, Maebashi, Gunma, 371-8511, Japan; 2 Department of Radiology, Maebashi Red Cross Hospital, Maebashi, Gunma, Japan

**Keywords:** Multidetector-row computed tomography, Maximum intensity projection, Celiac artery, Branching angle, Intra-arterial hepatic port system

## Abstract

**Background:**

We performed this study in order to investigate the shape of the origin of the celiac artery in maximum intensity projection (MIP) using routine 64 multidetector-row computed tomography (MDCT) data in order to plan for the implantation of an intra-arterial hepatic port system.

**Methods:**

A total of 1,104 patients with hepatocellular carcinoma were assessed with MDCT. In the definition of the branching angle, the anterior side of the abdominal aorta was considered the baseline, and the cranial and caudal sides were designated as 0 and 180 degrees, respectively. The angles between 0 and 90 degrees and between 90 and 180 degrees from the cranial side were considered upward and downward, respectively, and the branching angle of the celiac artery was classified every 30 degrees. The subclavian arterial route was used for the implantation of an intra-arterial hepatic port system in patients with branching angles of 150 degrees or more (sharp downward).

**Results:**

The median branching angle was (median ± standard deviation) 135 ± 23 (range, 51–174) degrees. The branching was upward in 77 patients (7%) and downward in 1,027 patients (93%). The branching was downward with an angle of 120 to150 degrees in most patients (n = 613). The branching was sharply downward with an angle of 150 degrees or more in 177 patients (16%). A total of 10 patients were referred for interventional placement of an intra-arterial hepatic port system. The subclavian arterial route was used for implantation of an intra-arterial hepatic port system in 2 patients with sharp downward branching.

**Conclusions:**

The branching angle of the celiac artery can be easily determined by the preparation of MIP images from routine MDCT data. MIP may provide useful information for the selection of the catheter insertion route in order to avoid a sharp branching angle of the celiac artery.

## Background

Besides a hepatectomy, systemic chemotherapy and arterial chemoinfusion therapy are used to treat primary and liver metastatic cancers. Catheter insertion is necessary for arterial infusion chemotherapy, and there are surgical and percutaneous catheter insertion methods. The low invasiveness of catheter insertions is important. There have been many reports of percutaneous implantations of port-catheter systems, which are a superior choice as it is a less invasive method. Methods for the percutaneous implantation of a port-catheter system are roughly divided into the subclavian arterial route method and the femoral arterial/inferior epigastric arterial route method [1-4]. The catheter insertion route is selected depending on the branching angle (upward or downward) of the origin of the celiac artery in some cases, and assessments of the branching angle before catheter insertion may increase the reliability of the technique. There have been no reports in the English literature about the branching angle of the celiac artery.

In this study, we analyzed the branching angle of the celiac artery using multidetector-row computed tomography (MDCT) in the planning of radiological catheter placement for the implantation of an intra-arterial hepatic port system. We prepared a maximum intensity projection (MIP) of a multiple projection volume reconstruction (MPVR) from the volume data of 64 MDCT with regard to the shape (upward or downward) of the origin of the celiac artery in patients who underwent routine MDCT [5,6] and investigated prior evaluations of the shape of the celiac artery.

### Patients and methods

#### Patients

Computed tomography (CT) was performed in 1,800 patients with hepatocellular carcinoma from Jan 1st, 2008 to Dec 31, 2010. The study was performed in 1,200 patients who were aged 19–91 years (median age: 62 years), and there were 687 males and 513 females. The patients were satisfied the following inclusion criteria: (a) contrast CT was done; (b) the origin of the celiac artery was in the imaged region; (c) no implantation of a port-catheter system had been performed; (d) the vascular anatomy was not of the celiacomesenteric type; (e) no history of abdominal operations; and (f) written informed consent was obtained. This study was approved by the institutional review board at Gunma University. This study was conducted in accordance with the amended Helsinki Declaration.

#### CT Technique

Routine CT was performed in all patients on MDCT (Aquilion 64, Toshiba Medical Systems Corporation, Tokyo, Japan) after mechanical injection (AutoenhanceA-250, Nemoto Kyorindo Co., Ltd., Tokyo, Japan) of l00–150 mL of nonionic iodinated contrast medium with a concentration of 300 mg/mL [Iopamiron300 (iopamidol), Nihon Shering K.K., Osaka, Japan, and Omunipaque 300 (iohexol), Daiichi Sankyo Co., Ltd., Tokyo, Japan]. The medium was administered at a dose of 2 mL/kg bodyweight to a maximum of 150 mL. The contrast material was injected at a rate of 1.5–4 mL/s through a 20- or 21-gauge intravenous cannula. The examinations were performed in a cephalocaudal direction starting at the top of the liver, and each examination included non-enhanced scanning and contrast-enhanced scanning in 3 phases: a hepatic-arterial phase, a portal venous phase and an equilibrium phase. The hepatic arterial phase was started 35 s after the start of injection, the portal phase at 70 s, and the equilibrium phase at 140 s.

Scanning was acquired during inhalation in all patients. The volume data that were obtained were reconstructed by setting the slice thickness to the collimation thickness (1 or 2 mm), the reconstruction field of view (FOV) to 150 mm, and the reconstruction interval to 1/2 of the collimation thickness (0.5 or 1 mm).

#### Image processing

The axial raw data images were processed on a commercially available image processing workstation (ZIO M900 QUADRA, Amin Co., Ltd., Tokyo, Japan). Vascular maps were generated from the processed axial date using MIP. The axial view at the level of the origin of the celiac artery was prepared, and a MPVR with the width of the abdominal aorta was prepared. The direction of the axial image was set to the relative direction of the region from the origin of the celiac artery to the branching of the hepatic and splenic artery. The branching angle of the origin of the celiac artery was measured in the MPVR image of the sagittal MIP.

#### Image analysis

**Figure 1:**
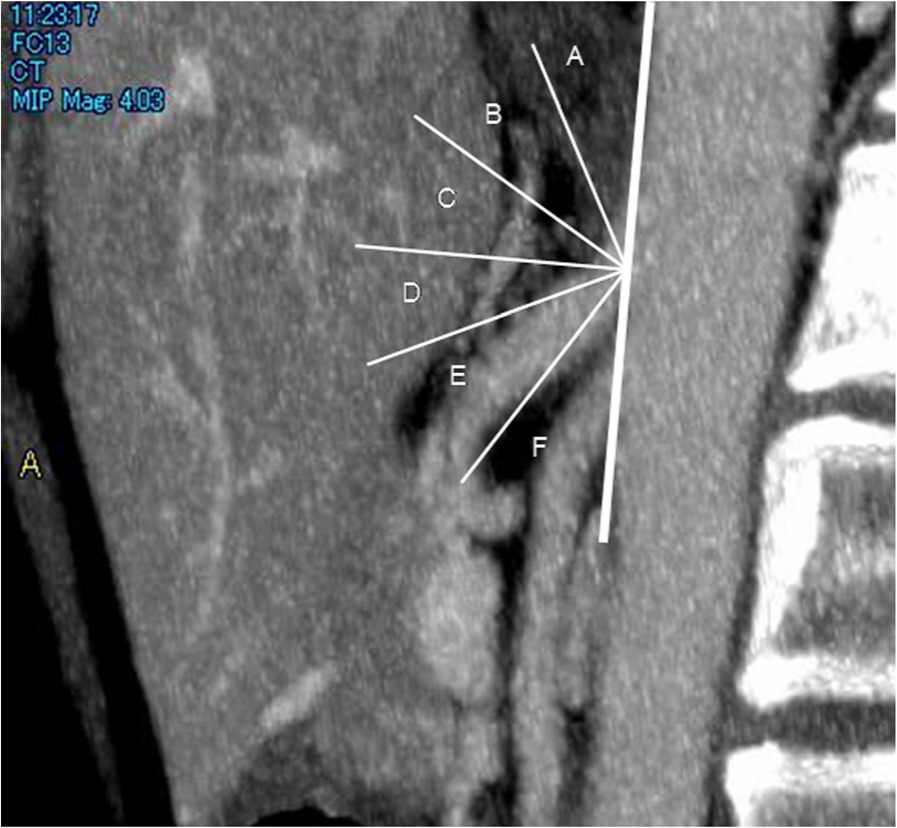
**Classification of the branching angle.** The anterior side of the abdominal aorta was regarded as the baseline, and the cranial and caudal sides were designated as 0 and 180 degrees, respectively. The branching angles between 0 and 90 and 180 degrees from the cranial side were regarded as upward and downward, respectively, and the celiac arterial branching angle was classified every 30 degrees.

In the definition of the branching angle, the anterior side of the abdominal aorta was regarded as the baseline, and the cranial and caudal sides were designated as 0 and 180 degrees, respectively. The angles between 0 and 90 degrees and between 90 and 180 degrees from the cranial side were regarded as upward and downward, respectively, and the branching angle of the celiac artery was classified every 30 degrees. Cases that were difficult to evaluate were presented as not evaluable (NE). The branching angle of the celiac artery was investigated based on this classification method (Figure 1).

#### Port implantation

The subclavian arterial route was used for implantation of an intra-arterial hepatic port system in patients with branching angles of 150 degrees or more (sharp downward). The femoral arterial route was used in patients with branching angles of 149 degrees or less. Technical success was defined as implantation of the catheter and port system.

Informed consent was obtained from each patient before of an intra-arterial hepatic port system was performed.

## Results

The subjects were 1,200 patients. The branching angle of the origin of the celiac artery was measured in the MPVR of the sagittal MIP. All patients had hepatocellular carcinoma. Analysis and evaluation of the branching angle was difficult in 96 patients (8%) who were then excluded and presented as NE. The details of these NE cases are as follows: (a) allergic symptoms developed immediately after the administration of contrast medium, the images were acquired after treatment, or evaluation was difficult because of poor contrast: 3 patients; (b) identification of the branching site was difficult because of severe narrowing of the origin of the celiac artery: 31 patients; and (c) evaluation of the branching angle was difficult because of severe calcification of the origin of the celiac artery: 62 patients.

**Figure 2:**
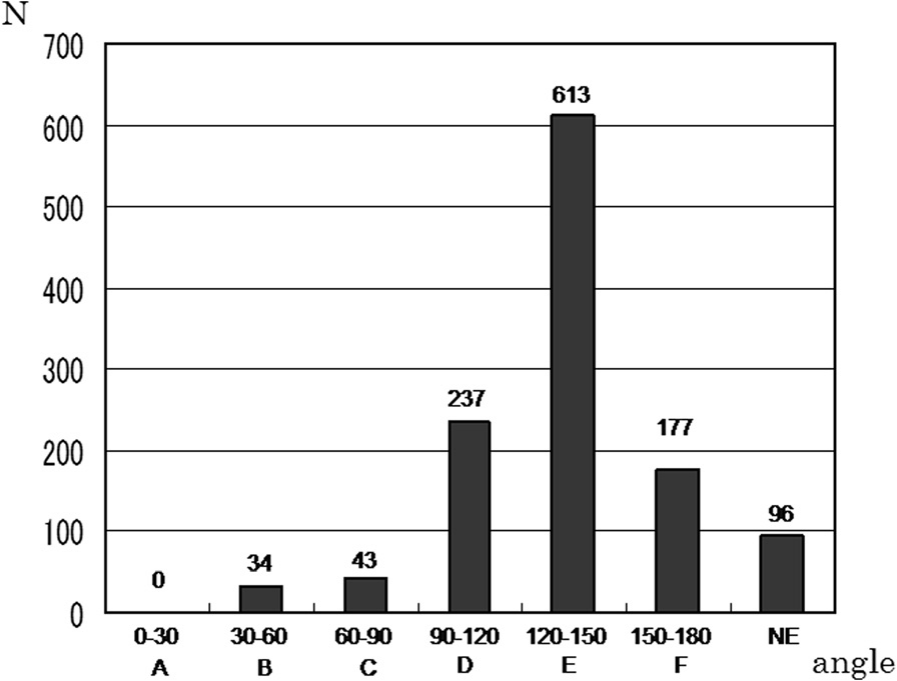
**A total of 1,200 patients with hepatocellular carcinoma underwent multidetector-row computed tomography.** The branching angle of the celiac artery was classified every 30 degrees. NE: not evaluable, N: number.

Analysis of the branching angle of the origin of the celiac artery was possible in 1,104 patients. The median branching angle was (median ± SD) 135 ± 23 (range, 51–174) degrees in these cases. These patients were divided into 6 groups. No patient was included in Group A, which represented cases with sharp upward branching with an angle less than 30 degrees, and 77 patients (7%) were included in Groups B and C, which represented cases with upward branching with an angle less than 90 degrees. The artery branched downward in most patients, and the branching was sharp downward with an angle of 150 degrees or more in 177 patients (16%) in group F (Figure 2).

**Figure 3:**
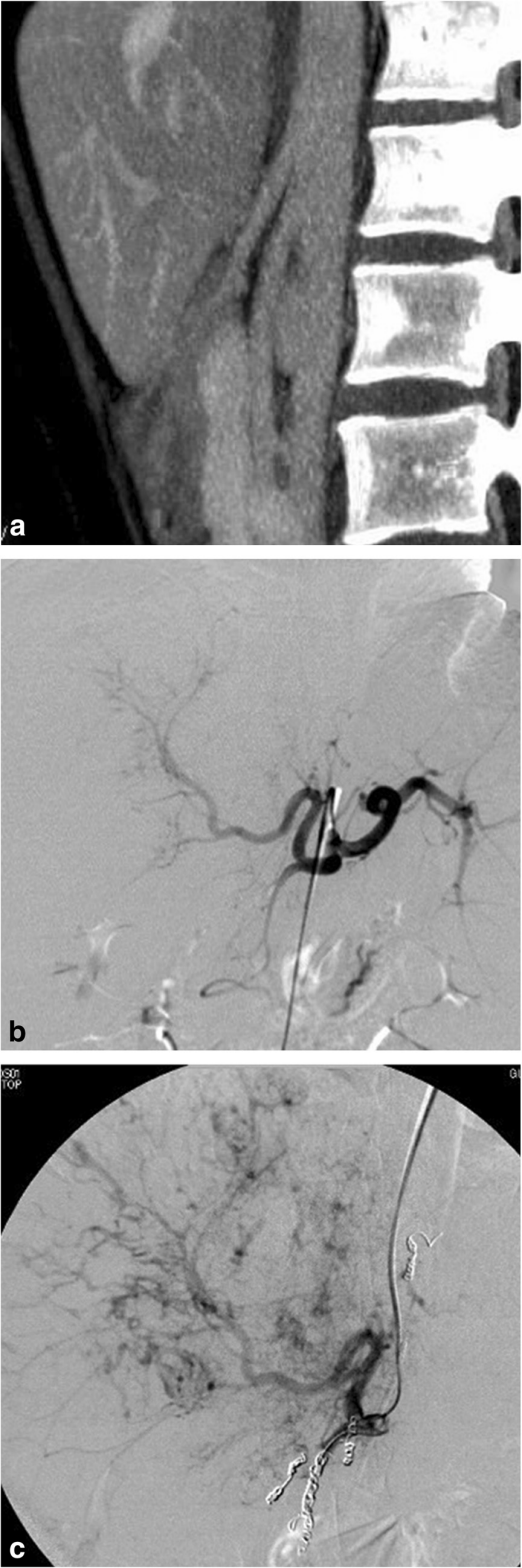
**A case of a 58-year-old woman with hepatocellular carcinoma.** The branching angle of the origin of the celiac artery was 168 degrees in the multiple projection volume reconstruction image of the sagittal maximum intensity projection (**a**). Digital subtraction angiography showed sharp downward branching of the celiac artery (**b**). The subclavian arterial route was selected for implantation of an intra-arterial hepatic port system (**c**).

A total of 10 patients were referred for interventional placement of an intra-arterial hepatic port system. There were no patients who were referred for interventional management in the NE cases. Technical success was 100% without any major adverse events that were associated with the procedure. The subclavian arterial route was used for the implantation of an intra-arterial hepatic port system in 2 patients with sharp downward branching (Figures 3a, b, and c). The femoral arterial route was used in the other 8 patients with branching angles of 149 degrees or less.

## Discussion

This study showed that MDCT provides useful information of the branching angle of the celiac artery for selection of the catheter insertion route.

Less invasive implantation of a port-catheter system is important, and percutaneous insertion is recommended. The catheter insertion methods are roughly divided into the subclavian arterial route method and the femoral arterial/inferior epigastric arterial route method. The passing of large joints, such as the hip and shoulder joints, complications, such as cerebral infarction, and the skill of the operator are factors that are important in selecting the insertion method. However, in order to complete arterial chemoinfusion therapy, stable catheter insertion is necessary, and the branching angle of the celiac artery may contribute to the stability. Thus, MIP was prepared from routine MDCT data in order to investigate the branching angle of the origin of the celiac artery. Although breathing-induced changes were not investigated in this study, the celiac artery branched downward in 1,027 of the 1,104 patients analyzed (more than 90%). The median branching angle was 135 degrees, and the artery branched downward sharply with an angle of 150 degrees or more in 177 patients.

Evaluation was difficult in 96 patients, and the branching angle could not be measured in these patients. The number of patient excluded from analysis may be a high number in spite of using a 64 MDCT. The reason is unclear. There might be many patients who were affected by arteriosclerosis. However, the branching direction of the celiac artery was roughly confirmed. The branching was downward in most of these patients. If evaluation of the celiac angle was difficult, it may not affect the success of the implantation of the hepatic port system and the outcome.

The celiac artery branched downward in most cases, and compression of the celiac artery by the median arcuate ligament, which is located right above the celiac artery, may be one reason. Lindner and Kemprud reported that the celiac artery branched right below the median arcuate ligament in 25 of 75 autopsy cases (33%). The rate of downward direction of the celiac artery may be high due to compression by this ligament [7,8]. In addition, breathing affects the angle of celiac artery. CT scans were obtained during deep inspiration, and, thus, the angle of the celiac artery may be exaggerated. However, there have been no reports in the English literature about the branching angle of the celiac artery.

As shown by this study, the celiac artery may branch downward sharply. For selection of the catheter insertion method of percutaneous implantation of a port-catheter system, the branching angle of the origin of the celiac artery should be considered. This branching angle can be readily investigated by preparation of sagittal MIP from routine MDCT data. The course of the celiac axis affects the technique of catheter placement. When a transfemoral or transepigastric approach is used, a caudal course of the celiac axis tends to be more difficult than a cranial course because multiple inflection points result in a reduction in the torque of the catheter and the guide wire [9].

The limitations of our study include the insufficient long-term follow up. In addition, we did not show if the imaging technique is actually useful for enhancing the outcome or success rates, and we did not compare or contrast our technique with existing solutions, such as ultrasound. At our institute, contrast-enhanced CT is routinely used for the pretreatment evaluation of malignant hepatic tumors, and additional MDCT in the same study was considered to be adequate. We suggest that MDCT is accurate in the detection of abdominal arterial anatomy, variations, and abnormalities. In addition, CT provides information about the number, size, and location of hepatic tumors and the presence of an extrahepatic disease. These are advantages over ultrasound and other imaging methods. A longer follow-up period in a larger sample of patients would improve the evidence for the efficacy. However, prior information of the branching angle before catheter insertion may increase the reliability of the insertion technique and the completion rate of the therapy.

Imaging of the celiac artery can make the process of implantation of hepatic port systems less invasive by enabling a percutaneous catheter insertion method and thereby avoiding surgery.

## Conclusion

The branching angle of the celiac artery can be easily determined by the preparation of MIP images from routine MDCT data. MIP may provide useful information for the selection of the catheter insertion route in order to avoid sharp branching angles of the celiac artery.

## Competing interests

The authors declare that they have no competing interests.

## Authors’ contributions

HT participated in the design of the study and carried out the clinical examination. All authors read and approved the final manuscript.

## Pre-publication history

The pre-publication history for this paper can be accessed here:

http://www.biomedcentral.com/1471-2342/12/36/prepub
